# Efficient Lead Finding, Activity Enhancement and Preliminary Selectivity Control of Nuclear Receptor Ligands Bearing a Phenanthridinone Skeleton

**DOI:** 10.3390/ijms19072090

**Published:** 2018-07-18

**Authors:** Yuko Nishiyama, Shinya Fujii, Makoto Makishima, Yuichi Hashimoto, Minoru Ishikawa

**Affiliations:** 1 Institute for Quantitative Biosciences, The University of Tokyo, 1-1-1 Yayoi, Bunkyo-ku, Tokyo 113-0032, Japan; ynishiya@ncc.go.jp (Y.N.); fujiis@iam.u-tokyo.ac.jp (S.F.); hashimot@iam.u-tokyo.ac.jp (Y.H.); 2 Nihon University School of Medicine, 30-1 Oyaguchi-kamicho, Itabashi-ku, Tokyo 173-8610, Japan; makishima.makoto@nihon-u.ac.jp

**Keywords:** phenanthridinone, steroid surrogate, antagonist

## Abstract

Background: Nuclear receptors (NRs) are considered as potential drug targets because they control diverse biological functions. However, steroidal ligands for NRs have the potential to cross-react with other nuclear receptors, so development of non-steroidal NR ligands is desirable to obtain safer agents for clinical use. We anticipated that efficient lead finding and enhancement of activity toward nuclear receptors recognizing endogenous steroidal ligands might be achieved by exhaustive evaluation of a steroid surrogate library coupled with examination of structure-activity relationships (SAR). Method: We evaluated our library of RORs (retinoic acid receptor-related orphan receptors) inverse agonists and/or PR (progesterone receptor) antagonists based on the phenanthridinone skeleton for antagonistic activities toward liver X receptors (LXRs), androgen receptor (AR) and glucocorticoid receptor (GR) and examined their SAR. Results: Potent LXRβ, AR, and GR antagonists were identified. SAR studies led to a potent AR antagonist (IC_50_: 0.059 μM). Conclusions: Our approach proved effective for efficient lead finding, activity enhancement and preliminary control of selectivity over other receptors. The phenanthridinone skeleton appears to be a promising steroid surrogate.

## 1. Introduction

**Figure 1:**
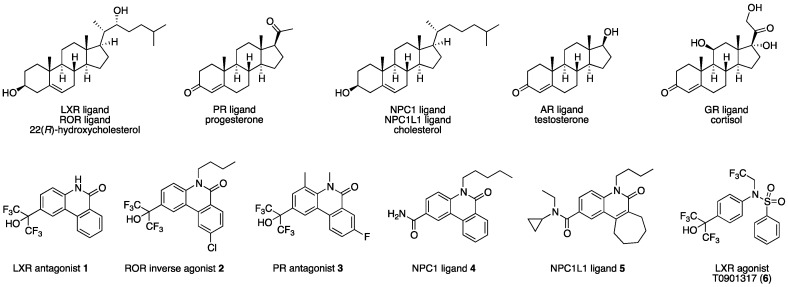
Chemical structures of endogenous steroidal ligands for NRs and phenanthridinone ligands for target proteins that recognize endogenous steroidal ligands.

Nuclear receptors (NRs) are ligand-dependent transcription factors that regulate DNA transcription by binding small molecular agonists such as hormones. Forty-eight structurally conserved kinds of NRs have been identified, and many of them recognize endogenous steroidal ligands (Figure 1). Because NRs control diverse biological functions including reproduction, differentiation, homeostasis, and the immune system, they were the targets of approximately 5% of FDA-approved drugs in 2011 [1]. However, not only a steroidal ligand, but also its metabolites, brings with it the potential to cross-react with other nuclear receptors, which can result in unwanted side effects, potentially limiting the clinical utility of these agents. Therefore, surrogates for the steroid skeleton are required to develop selective non-steroidal NR ligands for clinical use [2].

The number of fold structures of human proteins is at least 50 times smaller than the number of human proteins [2]. This fact indicates that a scaffold that is spatially complementary to one-fold structure might serve as a common scaffold for ligands that would interact specifically with more than 50 different human proteins, neglecting interactions of the peptide sequences of the proteins. In other words, the structures of ligands that bind to one member of fold structures may be useful for the development of novel lead compounds for other proteins with the similar fold structure. However, lead compounds obtained based on this approach are likely to possess polypharmacological character. Therefore, chemical modification of polypharmacological lead compounds, aimed at optimizing selectivity for a particular target, is required. Since the fold structures of NR ligand-binding domains (LBDs) are similar, we have used this strategy to create ligands bearing a diphenylmethane skeleton for several NRs, including farnesoid X receptor [3], liver X receptor (LXR) [4], vitamin D receptor [5], androgen receptor (AR) [6], and estrogen receptor [7].

We have previously developed LXRs antagonist **1** [8], retinoic acid receptor-related orphan receptors (RORs) inverse agonist **2** [9], progesterone receptor (PR) antagonist **3** [10], and pharmacological chaperones **4** and **5** for Niemann-Pick disease type C1 (NPC1) [11] and Niemann-Pick type C1-like 1 (NPC1L1) (Figure 1) [12]. These compounds all contain a phenanthridin-6(5*H*)-one skeleton as a cyclized carba-analog of the skeleton of LXRs pan agonist T0901317 (**6**). Because endogenous ligands for the above proteins (LXRs, RORs, PR, NPLC1 and NPC1L1) all have a steroid skeleton, we hypothesized that if the phenanthridin-6(5*H*)-one scaffold acts as a steroid surrogate, phenanthridinone derivatives would also bind to other NRs that recognize endogenous steroidal ligands. The approach described here is expected to be useful in the development of novel steroid surrogates, such as the phenanthridinone skeleton, enabling efficient lead finding of ligands for target proteins that recognize endogenous steroidal ligands, and the generation of more selective ligands by further molecular modifications of new and convenient scaffolds (easy to synthesis, easy to introduce substitutent(s) at any position, etc.), compared to the steroid skeleton. Here, we show that application of this approach to our small library of phenanthridinone-based RORs inverse agonists and PR antagonists led to several selective LXRβ, AR and glucocorticoid receptor (GR) antagonists.

## 2. Results and Discussion

AR and GR, members of NRs, recognize endogenous steroidal ligands as shown in Figure 1. In addition, because LXRs antagonistic activities of only limited numbers of phenanthridinone analog have been reported [8], precise structure-activity relationships (SAR) remain unclear. Therefore, we exhaustively evaluated our small library of RORs inverse agonists and/or PR antagonists bearing a phenanthridinone skeleton for LXRα, LXRβ, AR and GR antagonistic activities, and examined their SAR. LXRs regulate ATP-binding cassette proteins (ABCs), ApoE and glucose transporter 4 (GLUT4), are involved in lipid metabolism, reverse cholesterol transport, and glucose transport, so LXRs agonists are likely to be of therapeutic value in the treatment of atherosclerosis, hyperlipidemia, and metabolic syndrome. They are also involved in the upregulation of sterol regulatory element-binding protein-1c (SREBP-1c) and fatty acid synthase (FAS), so LXRs antagonists might have therapeutic value for hepatic steatosis [13]. AR is a receptor of androgens essential for the development and maintenance of the male reproductive system and secondary male sex characteristics. AR antagonists including hydroxyflutamide (OHF) are used for treatment of androgen-dependent tumors, especially prostate tumors. GR is a receptor of cortisol, and selective antagonists of GR could be useful in treating hypercortisolemia associated with Cushing’s syndrome and other conditions in which the endogenous GR is hyperactivated either through higher glucocorticoid levels or increased receptor sensitivity [14]. A steroidal GR antagonist, mifepristone (RU486), shows potent activities toward other steroid receptors.

For this work, compounds **1**–**3**, **7**–**42** and **44** were prepared as described previously [9,10,15]. PR-antagonistic activity was evaluated by assay of PR-regulated alkaline phosphatase activity in human breast cancer cell line T47D [10,16] while RORα, RORβ and RORγ inverse agonistic activities, and LXRα, LXRβ, AR and GR antagonistic activities were evaluated by reporter gene assay in HEK293 cell line [9,10]. Concentration of the agonists were set as around their EC_50_ values. Reproducibility of our assay systems is shown in Table S1. First, we focused on the SAR at the 2-position of phenanthridinone. The results, including the activities of positive controls T0901317 (**6**), RU486, and OHF, are shown in Table 1. We have reported that introduction of alkyl groups (**8**–**10**) or a hydroxymethyl group (**11**) at the 2-position of **7** resulted in retention or decrease of the PR-antagonistic activity [10] and RORs inverse agonistic activity [9]. Introduction of a hexafluoropropanol moiety somewhat increased the RORs activity and PR activity. According to the reported X-ray crystal structures of compound **6** and human LXRs LBD, the hydroxyl group of **6** forms a hydrogen bond with a histidine residue (His421 for LXRα and His435 for LXRβ) in helix 11 [17,18]. The SARs for RORs and PR are broadly consistent with those of LXRs, AR, and GR. For AR and PR, introduction of a hydroxymethyl group (**11**) resulted in retention of the activity, whereas introduction of hexafluoropropanol (**12**) resulted in 6-fold and 9-fold increases of PR and AR activity, respectively. On the other hand, for LXRs and RORs, **11** showed weaker activity than **7** whereas **12** showed stronger activity than **7**, suggesting that the two trifluoromethyl groups might be associated with more potent activity and the hydroxyl group might not form a strong hydrogen bond with histidine in these receptors, in contrast to the other receptors. This idea is consistent with the reported co-crystal structure of T0901317 (**6**) complexed with RORγ [19]. Overall, we found that compound **12** showed not only PR-antagonistic activity and RORs inverse agonistic activity, but also LXRα, LXRβ, AR and GR antagonistic activity (Table 1). These results support our view that the phenanthridin-6(5*H*)-one scaffold acts as a steroid surrogate.

Next, SAR at the nitrogen atom is shown in Table 2. Similar to the SAR for RORs, introduction of a longer-chain alkyl group on the nitrogen atom (**12** and **15**) resulted in enhancement of the GR antagonistic activity. SAR for AR was similar to that for PR, that is, hydrogen analog **12** showed potent AR antagonistic activity with an IC_50_ value of 0.10 μM, and more than 200-fold selectivity for AR over RORs and GR, and about 7.8- and 50-fold selectivity over PR and LXRs, respectively. The longest alkyl analog **17** showed decreased activity toward all NRs, suggesting that the binding pocket hosting the N-alkylated derivatives might not be able to accommodate a group larger than a hexyl group.

Next, SAR at the nitrogen atom is shown in Table 2. Similar to the SAR for RORs, introduction of a longer-chain alkyl group on the nitrogen atom (**12** and **15**) resulted in enhancement of the GR antagonistic activity. SAR for AR was similar to that for PR, that is, hydrogen analog **12** showed potent AR antagonistic activity with an IC_50_ value of 0.10 μM, and more than 200-fold selectivity for AR over RORs and GR, and about 7.8- and 50-fold selectivity over PR and LXRs, respectively. The longest alkyl analog **17** showed decreased activity towards all NRs, suggesting that the binding pocket hosting the N-alkylated derivatives might not be able to accommodate a group larger than a hexyl group.

Next, the effect of introduction of a methoxy group at every position (methoxy scanning) was investigated (**18**–**24**; Table 3) because many types of NR ligands possess a methoxy group(s) [20]. We have reported that 3-methoxy and 4-methoxy analogs **19** and **20** showed more than 3-fold and 2-fold enhanced PR activity compared with the unsubstituted analog **12**, respectively [10]. In contrast, 9-methoxy analog **23** showed 5-fold weaker PR activity than **12**. In the case of RORs, we have reported that introduction of a methoxy group at the 9-position (**23**) enhanced ROR activities [9]. The activities toward LXRs, AR and GR also depended on the position of the methoxy group. 1-Methoxy analog **18** showed decreased activity for all receptors tested. Enhanced activity was observed when a methoxy group was introduced at the 4-, 9- or 10-position for LXRα, 9- or 10-position for LXRβ, 3- and 8-position for AR, and 3- or 4-position for GR. Especially, 3-methoxy analog **19** showed 5.7-fold improved GR activity compared with **12**. Overall, introduction of a methoxy group resulted in increased activity in almost all cases except RORβ, but the most suitable position depended on the NR (3-position: PR and GR, 8-position: AR, 9-position: RORα, RORγ and LXRβ, 10-position: LXRα). The introduction of a substituent at every position is useful to investigate substituent effects. In this context, it is important to note that phenanthridin-6(5*H*)-one analogs bearing substituent(s) at any position can be quite easily synthesized, and this represents a considerable advantage compared to the steroid skeleton.

The results of methoxy scanning prompted us to investigate the effect of introduction of two methoxy groups (**25**–**31** Table 3). In the cases of RORα, PR, AR and GR, 3,4-dimethoxy analog **25** exhibited similar or weaker activity to the 3-methoxy and 4-methoxy analogs **19** and **20**. A possible explanation might be the direction of the two methoxy groups, that is, one or both might occupy a space not suitable for receptor interaction due to steric hindrance. This idea is supported by the fact that 3,4-dioxolane analog **26** showed stronger activity than **25** toward PR, AR and GR. Compound **26** was the most potent GR antagonist with an IC_50_ value of 0.76 μM, although this compound also showed PR and AR antagonistic activities with IC_50_ values of 0.23 and 0.54 μM, respectively. When two methoxy groups were introduced at distal positions, the activity was improved. Thus, 3,8-dimethoxy analog **27** and 4,8-dimethoxy analog **28** showed more potent PR-antagonistic activity than monomethoxy analogs **19**, **20**, and **22**, and 3,8-dimethoxy analog **27** showed more potent AR antagonistic activity than monomethoxy analogs **19** and **22**.

We next examined the SAR of fluoro derivatives **32**–**35** (Table 4) to investigate the effect of this electron-withdrawing substituent. For PR, the preferences for fluorine substitution position were roughly consistent with the results of the methoxy scanning, that is, 4- and 8-substitued analogs **32** and **34** showed enhanced PR activity, whereas 9-substituted analogs **35** showed weaker PR activity [10]. For AR, all fluoro analogs **32**–**35** showed enhanced activity compared with **12**, suggesting that an electron-withdrawing effect at these positions is important for AR antagonistic activity. For LXRα, 4- and 7-fluoro analogs **32** and **33** showed enhanced activity compared with **12**.

We then examined various substituents at the 9-position (Table 5). We have reported that introduction of a chlorine atom at the 9-position (**2**) enhanced inverse agonistic activity toward RORγ [9]. The SARs of RORγ were roughly consistent with the results for LXRβ, and **2** also exhibited potent LXRβ antagonistic activity with the IC_50_ value of 0.88 μM. Replacement of fluorine at the 9-position (**35**) with chlorine (**2**) caused enhanced activity toward RORs and LXRβ, but decreased activity toward PR, AR, and GR, suggesting that molecular modification at the 9-position would be important to improve selectivity for metabolic NRs over steroid receptors.

**Scheme 1:**
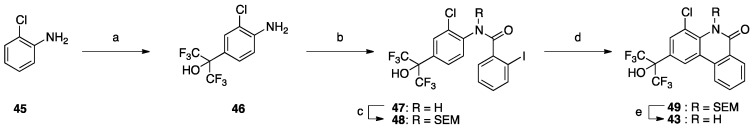
Reagents and conditions: (a) CF_3_COCF_3_·1.5H_2_O, *p*-TsOH·H_2_O, toluene, reflux, 27%; (b) 2-iodobenzoic acid, EDC, DMAP, DMF, 100 °C; (c) SEMCl, NaH, DMF, 0 °C to RT; (d) Pd(OAc)_2_, PCy_3_·HBF_4_, Cs_2_CO_3_, DMA, 130 °C, 24% in 2 steps; (e) TBAF, THF, reflux, 18%.

Next, we focused on the 4-position (Table 6), because 4-methoxy analog **20** showed enhanced RORα, LXRα and PR activity, and 4-fluoro analog **32** showed enhanced LXRα, PR and AR activity. Compounds **39**–**44** and **2** showed weak activity toward RORs and GR, indicating that the alkyl group on the nitrogen atom is important for activity toward these receptors. We have reported that 4-alkyl analogs **40**–**42** showed greater PR activity than the unsubstituted analog **1**, and 4-methyl analog **40** had an IC_50_ value of 0.15 µM [10]. As for LXRs and AR, 4-alkyl analogs **40**–**42** showed decreased activity compared with the unsubstituted analog **1**, whereas 4-fluoro analog **32** showed greater activity than **1**. Based on the reported SARs of non-steroidal AR antagonists, an electron-withdrawing substituent neighboring the amide bond is expected to be important for potent AR antagonism [21]. Therefore, 4-chloro analog **43** was designed and synthesized as shown in Scheme 1. Interestingly, **43** showed greater AR activity and weaker RORs, LXRs, PR and GR activity than 4-fluoro analog **32**. Compound **43** showed the IC_50_ value of 0.059 μM, being 2.9-fold more potent than clinically used OHF under our assay conditions. Compound **43** showed more than 340-fold selectivity over RORs and LXRβ, and 90-fold and 6-fold selectivity over LXRα and PR, respectively. This result indicates that molecular modification at the 4-position can increase selectivity for AR over the other NRs.

**Figure 2:**
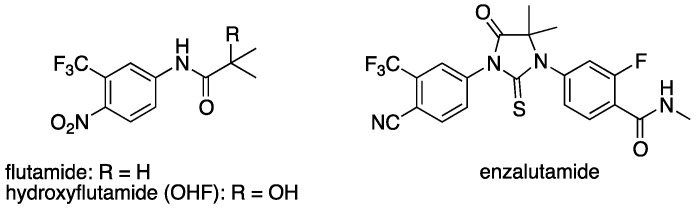
Chemical structures of AR antagonists.

Chronic administration of AR antagonists often leads to the development of resistance. Mutations in the LBD of AR, such as T877A, have been identified in patients treated with flutamide (Figure 2), and the activated metabolite, hydroxyflutamide, is an agonist of AR bearing T877A [22]. In contrast, another AR antagonist, enzalutamide, was reported to antagonize AR mutant T877A [23]. Thus, development of a novel chemical class of AR antagonists, different from flutamide analogs seems an attractive approach for the treatment of AR antagonist-resistant cancer, providing further motivation to evaluate steroid surrogates. Therefore, we investigated the antiandrogenic activity of several potent AR antagonists by means of PSA (prostate-specific antigen, an AR-regulated gene) ELISA (enzyme-linked immunosorbent assay) in human prostate cancer cell line LNCaP, which has T877A mutation in the AR LBD. Potent AR antagonist **43** did not show antiandrogenic activity in LNCaP, whereas several other AR antagonists including **33** decreased the PSA level with an IC_50_ of 190 nM (Table 7). This result suggests that the new non-flutamide type AR antagonist **33** is a promising candidate for antiandrogen therapy of prostate cancer.

## 3. Materials and Methods

### 3.1. Chemistry

#### 3.1.1. General

^1^H NMR spectra were recorded on a JNM-ECA500 (500 MHz) spectrometer (JEOL Ltd., Tokyo, Japan). Chemical shifts (δ) are reported in parts per million. Flash column chromatography was performed on silica gel 60N (40–50 mm) (Kanto Chemical Co., Inc., Tokyo, Japan). HPLC analyses to check purity were performed on an analytical column (GL Science Inc., Tokyo, Japan) Inertsil ODS-4 reversed-phase column, 5 μm, 4.6 mm × 150 mm) eluted with a mobile phase consisting of CH_3_CN/water at a flow rate of 1.0 mL/min, with UV (ultraviolet) monitoring at 254 nm, at 37 °C.

#### 3.1.2. 2-(4-Amino-3-chlorophenyl)-1,1,1,3,3,3-hexafluoropropan-2-ol (**46**)

To a solution of 2-chloroaniline (**45**) (1.58 mL, 15.0 mmol) in toluene (11 mL) were added hexafluoroacetone trihydrate (2.06 mL, 18.0 mmol) and *p*-TsOH (258 mg, 1.50 mmol). The mixture was stirred for 8.5 h at 110 °C, and then cooled to room temperature. Water was added, and the resulting mixture was extracted with AcOEt. The combined organic layer was washed with brine, dried over Na_2_SO_4_, and concentrated under reduced pressure. The residue was purified by silica gel column chromatography (*n*-hexane/AcOEt = 10:1-3:1) to give **46** as a pink solid (9%). ^1^H NMR (500 MHz, CDCl_3_) δ: 7.58 (s, 1H), 7.35 (d, *J* = 8.6 Hz, 1H), (d, *J* = 9.2 Hz, 1H).

#### 3.1.3. *N*-(2-Chloro-4-(1,1,1,3,3,3-hexafluoro-2-hydroxypropan-2-yl)phenyl)-2-iodobenzamide (**47**)

To a solution of EDC (742 mg, 3.87 mmol) and DMAP (473 mg, 3.87 mmol) in DMF (4.0 mL) was added 2-iodobenzoic acid (640 mg, 2.58 mmol) under an Ar atmosphere. The mixture was stirred for 30 min at room temperature, then **46** (379 mg, 1.29 mmol) was added to it, and stirring was continued at 100 °C for 4 h. The resulting mixture was cooled, diluted with water, and extracted with AcOEt. The combined organic layer was washed with brine, dried over Na_2_SO_4_, and concentrated under reduced pressure. The residue was purified by column chromatography (*n*-hexane/AcOEt = 4:1) to give **47** (181 mg, 0.346 mmol, 27%) as a brown solid. ^1^H NMR (500 MHz, DMSO-*d_6_*) δ: 7.94 (d, *J* = 8.0 Hz, 1H), 7.89 (d, *J* = 8.0 Hz, 1H), 7.76 (d, *J* = 1.7 Hz, 1H), 7.69 (d, *J* = 8.0 Hz, 1H), 7.52 (d, *J* = 4.6 Hz, 2H), 7.24 (td, *J* = 3.9, 1.3 Hz, 1H).

#### 3.1.4. *N*-(2-Chloro-4-(1,1,1,3,3,3-hexafluoro-2-((2-(trimethylsilyl)ethoxy)methoxy)propan-2-yl)phenyl)-2-iodo-*N*-((2-(trimethylsilyl)ethoxy)methyl)benzamide (**48**)

Sodium hydride (41.6 mg, 1.04 mmol) was added to a solution of **47** (181 mg, 0.346 mmol) in DMF (1.5 mL) at 0 °C. The mixture was stirred for 1 h at 0 °C, then SEMCl (184 μL, 1.04 mmol) was added at 0 °C, and stirring was continued for 6 h at room temperature. The resulting mixture was diluted with AcOEt, quenched with water, and extracted with AcOEt. The combined organic layer was washed with water, dried over Na_2_SO_4_, and concentrated. The residue was purified by column chromatography (*n*-hexane/AcOEt = 20:1 to 10:1) to give **48** (219 mg, 0.279 mmol) as a colorless oil. The compound was used for the next reaction without further purification.

#### 3.1.5. 4-Chloro-2-(1,1,1,3,3,3-hexafluoro-2-((2-(trimethylsilyl)ethoxy)methoxy)propan-2-yl)-5-((2-trimethylsilyl ethoxy)methyl)phenanthridin-6(5*H*)-one (49)

To a solution of **48** (219 mg, 0.279 mmol) in DMA (1.0 mL) were added PCy_3_·HBF_4_ (63.7 mg, 0.173 mmol), Cs_2_CO_3_ (620 mg, 1.90 mmol) and Pd(OAc)_2_ (12.0 mg, 0.0532 mmol) under an Ar atmosphere. The mixture was stirred for 3 h at 130 °C, then cooled to room temperature, and water was added. The resulting mixture was extracted with AcOEt. The combined organic layer was washed with brine, dried over Na_2_SO_4_, and concentrated under reduced pressure. The residue was purified by column chromatography (*n*-hexane/AcOEt = 20:1) to give **49** as a brown oil (45.2 mg, 0.0824 mmol, 24% in 2 steps). ^1^H NMR (500 MHz, CDCl_3_) δ: 8.56 (s, 1H), 8.53 (dd, *J* = 8.0, 1.1 Hz, 1H), 8.26 (d, *J* = 7.7, 1.5 Hz, 1H), 7.62 (t, *J* = 8.0 Hz, 1H), 7.47 (s, 1H), 5.85 (s, 2H), 4.95 (s, 2H), 3.88 (t, *J* = 8.6 Hz, 2H), 3.74 (t, *J* = 8.3 Hz, 2H), 1.02 (t, *J* = 8.3 Hz, 2H), 0.98 (m, 2H), 0.02 (s, 9H), −0.05 (s, 9H).

#### 3.1.6. 4-Chloro-2-(1,1,1,3,3,3-hexafluoro-2-hydroxypropan-2-yl)phenanthridin-6(5*H*)-one (**43**)

TBAF (1 M in THF, 0.779 mL, 0.779 mmol) was added to a solution of **49** (42.7 mg, 0.0779 mmol) in THF (0.8 mL) at room temperature. The reaction mixture was stirred under reflux for 9 h, then diluted with water, and extracted with AcOEt. The combined organic layer was washed with brine, dried over Na_2_SO_4_, and concentrated under reduced pressure. The residue was purified by column chromatography (CHCl_3_/MeOH = 40:1) to give **43** as a pale yellow solid (18%).

^1^H NMR (500 MHz, DMSO-*d_6_*) δ: 8.35 (d, *J* = 8.6 Hz, 1H), 8.27 (dd, *J* = 8.0, 1.1 Hz, 1H), 7.86–7.83 (m, 1H), 7.68 (d, *J* = 8.6 Hz, 1H), 7.63 (t, *J* = 7.4 Hz, 1H), 7.41 (d, *J* = 8.6 Hz, 1H). HLPC purity 97.0% (area %).

### 3.2. Biology

#### 3.2.1. T-47D Alkaline Phosphatase Assay

T-47D alkaline phosphatase assays were performed as described in the literature [10]. Briefly, human ductal breast epithelial tumor (T-47D) cells were treated with fresh medium containing test compound plus progesterone (final concentration 1 nM), and incubated for 24 h. The fixed cells were washed with PBS and assay buffer was added. After the reaction was terminated, the absorbance at 405 nm was measured. All data points were measured in triplicate.

#### 3.2.2. Reporter Gene Assay

Human embryonic kidney (HEK) 293 cells (RIKEN BRC, Ibaraki, Japan) were maintained in DMEM (Dulbecco’s Modified Eagle’s medium) containing 5% (*v*/*v*) fetal bovine serum, and penicillin/streptomycin at 37 °C in a humidified atmosphere of 5% CO_2_ in air. Cells were seeded in clear-bottomed white 96-well plates at a density of 1 × 10^4^ cells/well and incubated for 6 h prior to transfection. Cells were co-transfected with 30 ng of a NR expression plasmid, 50 ng of a luciferase reporter and 10 ng of CMX-β-galactosidase expression vector per well, using the calcium phosphate co-precipitation method. After 24 h, transfected cells were treated with dimethyl sulfoxide (DMSO) or DMSO solution of test compounds for 24 h. T0901317 (0.3 μM), T0901317 (0.1 μM), dihydrotestosterone (0.3 nM), and dexamethasone (1 nM) were used as agonists for LXRα, LXRβ, AR and GR, respectively. After addition of a luciferase substrate, luminescence was detected with a luminometer. The luciferase activity of each sample was normalized by β-galactosidase activity: 2-nitrophenyl-β-d-galactopyranoside was added, and the absorbance was measured at 405 nm. Each sample was carried out in triplicate. A six-point sigmoidal dose-response curve was generated for each compound. The IC_50_ value for each compound was calculated by linear approximation of two points adjacent to 50% inhibition with logarithmic scale. In Table S1, biological reproducibility are reported as the mean IC_50_ ± SEM.

Nuclear receptor plasmids: GR (CMX-hGR), AR (CMX-hAR), ROR (pcDNA3.1(-)-hRORα1, pcDNA3.1(-)-hRORβ1, pcDNA3.1(-)-hRORγ1), LXR (CMX-GAL4N-hLXRα-LBD, CMX-GAL4N-hLXRβ-LBD).

Luciferase reporter plasmids: GR (MTV-Luc), AR (ARE-Luc), ROR (RORE-TK-Luc), LXR (TK-MH100x4-Luc).

Plasmids for AR, GR and LXRs were provided by Prof. Dr. Makishima, and plasmids for RORs were provided by Itsuu Institute (Kanagawa, Japan).

#### 3.2.3. PSA Level in LNCaP Cell Line

The human prostate cancer cell line LNCaP (ECACC, London, UK) was routinely maintained in RPMI1640 containing 10% (*v*/*v*) fetal bovine serum, and penicillin/streptomycin at 37 °C in a humidified atmosphere of 5% CO_2_ in air. Cells were cultured in RPMI1640 without phenol red containing 10% (*v*/*v*) charcoal-stripped fetal bovine serum, and penicillin/streptomycin at 37 °C in a humidified atmosphere of 5% CO_2_ in air for 3 days. Then cells were plated in 96-well plates at 1 × 10^4^ cells/well and incubated overnight. On the next day, cells were treated with fresh media containing test compound plus dihydrotestosterone (final concentration 10 nM), and incubated for 48 h. Then, PSA levels were measured in the culture supernatants, after 100-fold dilution with the medium, using ELISA (Immunospec Corp., CA, USA Free prostate-specific antigen cat. #E29-212) according to the manufacturer’s instructions. All data points were carried out twice in triplicate.

## 4. Conclusions

Development of non-steroidal NR ligands is desirable to obtain safer, more selective agents for clinical use. Here, non-steroidal antagonists for multiple target proteins including LXRβ (IC_50_: 0.88 μM), AR (IC_50_: 0.10 μM), GR (IC_50_: 0.76 μM) were efficiently identified by exhaustive evaluation of a small steroid surrogate library. In addition, a potent AR antagonist **43** (IC_50_: 0.059 μM) belonging to a new chemical class was developed. Further, AR antagonist **33** showed antiandrogenic activity toward AR antagonist-resistant cell line LNCaP with the IC_50_ value of 190 nM. Structure-activity relationship studies of phenanthridinone analogs revealed that (1) hydrogen at the nitrogen is important for AR and PR activity, and a longer alkyl group is favorable for RORs and GR activity, (2) molecular modification at the 9-position can increase selectivity for metabolic NRs over steroid receptors, and (3) an electron-withdrawing substituent on the phenyl group is important for AR antagonistic activity, while modification at the 4-position can increase selectivity for AR over other NRs. The phenanthridinone skeleton is more readily modifiable than the steroid skeleton. Thus, our strategy of efficient lead finding, activity enhancement and preliminary control of selectivity over other receptors should expand the utility of the multi-template approach, even though the compounds obtained here still require further optimization.

## Figures and Tables

**Table 1:**
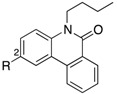
SAR at 2-position.

Compound	R	RORα ^1^	RORβ ^1^	RORγ ^1^	LXRα ^2^	LXRβ ^2^	PR ^2,3^	AR ^2^	GR ^2^
**6**	-	>20,000	>20,000	6500	290 ^4^	130 ^4^			
RU486	-						0.073		2.0
OH-Flu	-							170	
**7**	H	10,000	13,000	15,000	19,000	15,000	7100	17,000	>20,000
**8**	Me	18,000	17,000	17,000	17,000	16,000	7900	11,000	>20,000
**9**	Et	9000	7900	9200	17,000	16,000	7800	19,000	19,000
**10**	*t*-Bu	12,000	13,000	10,000	>20,000	>20,000	8300	17,000	>20,000
**11**	CH_2_OH	>20,000	>20,000	>20,000	>20,000	>20,000	8600	9600	>20,000
**12**	(CF_3_)_2_COH	9800	6500	3900	8400	3700	1200	1800	7400

^1^ Inverse agonistic activity (IC_50_: nM) [9]. ^2^ Antagonistic activity (IC_50_: nM). ^3^ [10]. ^4^ Agonistic activity (IC_50_: nM).

**Table 2:**
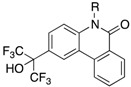
SAR at nitrogen atom.

Compound	R	RORα ^1^	RORβ ^1^	RORγ ^1^	LXRα ^2^	LXRβ ^2^	PR ^2,3^	AR ^2^	GR ^2^
**1**	H	>20,000	>20,000	>20,000	5000 ^4^	5100 ^4^	780	100	>20,000
**13**	Me	>20,000	>20,000	>20,000	4100 ^4^	>20,000 ^4^	340	500	>20,000
**14**	Et	>20,000	>20,000	>20,000	14,000	6600	1400	1700	>20,000
**15**	*n*-Pr	11,000	8200	4200	12,000	6900	3800	5600	17,000
**16**	*n*-Hex	7600	5400	4700	16,000	10,000	2500	4000	>20,000
**17**	*n*-C_9_H_19_	>20,000	>20,000	>20,000	>20,000	>20,000	10,000	15,000	>20,000

^1^ Inverse agonistic activity (IC_50_: nM) [9]. ^2^ Antagonistic activity (IC_50_: nM). ^3^ [10]. ^4^ [8].

**Table 3:**
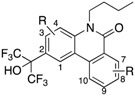
SAR of substitution effect of alkoxy groups.

Compound	R	RORα ^1^	RORβ ^1^	RORγ ^1^	LXRα ^2^	LXRβ ^2^	PR ^2,3^	AR ^2^	GR ^2^
**18**	1-OMe	>20,000	>20,000	>20,000	>20,000	>20,000	4000	>20,000	>20,000
**19**	3-OMe	8200	8200	7500	8400	9200	410	1100	1300
**20**	4-OMe	7500	6900	5400	6900	5500	560	2400	3900
**21**	7-OMe	12,000	15,000	13,000	11,000	12,000	12,000	9400	14,000
**22**	8-OMe	12,000	15,000	13,000	12,000	11,000	1100	1000	8200
**23**	9-OMe	6600	7200	2700	7000	2400	5800	>20,000	10,000
**24**	10-OMe	7700	>20,000	8500	4600	4300	2700	>20,000	>20,000
**25**	3,4-diOMe	>10,000	>10,000	8500	8500	9600	510	6100	>20,000
**26**	3,4-(OCH_2_O)	>20,000	>20,000	>20,000	>20,000	>20,000	230	540	760
**27**	3,8-diOMe	9500	13,000	12,000	17,000	18,000	300	580	1700
**28**	4,8-diOMe	11,000	11,000	9700	>10,000	>10,000	340	1700	5200
**29**	7,8-diOMe	7300	7700	8000	3200	3200	4200	4600	7900
**30**	7,9-diOMe	>20,000	>20,000	>20,000	>20,000	>20,000	>20,000	>20,000	>20,000
**31**	8,9-diOMe	18,000	16,000	19,000	8800	8900	9600	1600	17,000

^1^ Inverse agonistic activity (IC_50_: nM) [9]. ^2^ Antagonistic activity (IC_50_: nM). ^3^ [10].

**Table 4:**
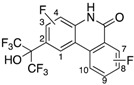
SAR of substitution effect of fluorine atom.

Compound	Position	RORα ^1^	RORβ ^1^	RORγ ^1^	LXRα ^2^	LXRβ ^2^	PR ^2,3^	AR ^2^	GR ^2^
**32**	4	15,000	14,000	18,000	3100	4600	130	390	6900
**33**	7	7900	9800	13,000	3100	3100	730	460	9800
**34**	8	17,000	11,000	>20,000	7900	6000	310	680	>20,000
**35**	9	9800	8300	5000	6400	5400	1000	920	7000

^1^ Inverse agonistic activity (IC_50_: nM) [9]. ^2^ Antagonistic activity (IC_50_: nM). ^3^ [10].

**Table 5:**
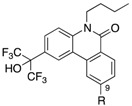
SAR of substitution effect at 9-posiiton.

Compound	R	RORα ^1^	RORβ ^1^	RORγ ^1^	LXRα ^2^	LXRβ ^2^	PR ^2,3^	AR ^2^	GR ^2^
**36**	Me	7600	7100	1000	12,000	3000	3700	6400	16,000
**2**	Cl	5600	4900	690	9200	880	4300	5200	10,000
**37**	CF_3_	5500	5600	2600	7100	4500	4200	>20,000	>20,000
**38**	OH	6400	5500	4100	7000	5500	5100	>3000	>20,000

^1^ Inverse agonistic activity (IC_50_: nM) [9]. ^2^ Antagonistic activity (IC_50_: nM). ^3^ [10].

**Table 6:**
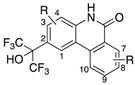
SAR at 4-posiiton and other substitution effects.

Compound	R	RORα ^1^	RORβ ^1^	RORγ ^1^	LXRα ^2^	LXRβ ^2^	PR ^2,3^	AR ^2^	GR ^2^
**39**	4-OMe	>20,000	>20,000	9600	>20,000	>20,000	270	>20,000	>20,000
**40**	4-Me	>20,000	>20,000	>20,000	>20,000	>20,000	150	>20,000	>20,000
**41**	4-Et	>20,000	>20,000	>20,000	>20,000	>20,000	210	14,000	>20,000
**42**	4-*n*-Pr	>20,000	11,000	9900	>20,000	20,000	360	5400	16,000
**43**	4-Cl	>20,000	>20,000	>20,000	5300	>20,000	350	59	>20,000
**44** ^3^	4-Me, 8-F	>20,000	>20,000	>20,000	>20,000	>20,000	46	1700	>20,000
**3** ^3^	-	>20,000	>20,000	>20,000	>20,000	>20,000	27	300	>20,000

^1^ Inverse agonistic activity (IC_50_: nM) [9] ^2^ Antagonistic activity (IC_50_: nM). ^3^ [10].

**Table 7:** Antiandrogenic activity toward AR antagonist-resistant cell line LNCaP.

Compound	AR Reporter Gene IC_50_ (nM)	LNCaP IC_50_ (nM) ± SEM
**1**	100	>10,000
**13**	500	>10,000
**26**	540	560 ± 260 (*N* = 2)
**32**	390 ± 30 (*N* = 2)	490 ± 260 (*N* = 2)
**33**	460	190 ± 95 (*N* = 2)
**34**	680	>10,000
**35**	920	550 ± 110 (*N* = 2)
**43**	59 ± 9.5 (*N* = 2)	>10,000

Mean IC_50_ values with standard error of mean (SEM) from 1 or N times independent experiments.

## Supplementary Materials

Supplementary materials can be found at http://www.mdpi.com/1422-0067/19/7/2090/s1.

## Abbreviations

NR	Nuclear receptors
SAR	Structure-activity relationship
PR	Progesterone receptor
LXR	Liver X receptor
AR	Androgen receptor
GR	Glucocorticoid receptor
LBD	Ligand-binding domains
ROR	retinoic acid receptor-related orphan receptors
NPC1	Niemann-Pick disease type C1
NPC1L1	Niemann-Pick disease type C1-like 1
OHF	Hydroxyflutamide
Ts	p-Toluenesulfonyl
EDC	*N*-Ethyl-*N*’-(3-dimethylaminopropyl)carbodiimide
DMAP	4-(*N*,*N*-Dimethylamino)pyridine
DMF	Dimethylformamide
SEM	2-(Trimethylsilyl)ethoxymethyl
Cy	Cyclohexyl
DMA	Dimethylacetamide
TBAF	Tetrabutylammonium fluoride
THF	Tetrahydrofuran
