# Supplementary material for: Efficient Lead Finding, Activity Enhancement and Preliminary Selectivity Control of Nuclear Receptor Ligands Bearing a Phenanthridinone Skeleton
Source: Int J Mol Sci. 2018 Jul 18;19(7):2090. doi: 10.3390/ijms19072090 (PMC6073568; doi:10.3390/ijms19072090)
Supplement: Supplementary file 1 [file ijms-19-02090-s001.pdf]

Supporting Information

Efficient lead finding, activity enhancement and preliminary selectivity control of nuclear receptor ligands bearing a phenanthridinone skeleton

Yuko Nishiyama [1], Shinya Fujii [1], Makoto Makishima [2], Yuichi Hashimoto [1], and Minoru Ishikawa [1,*]

[1] Institute for Quantitative Biosciences, The University of Tokyo, 1-1-1 Yayoi, Bunkyo-ku, Tokyo 113-0032, Japan

[2] Nihon University School of Medicine, 30-1 Oyaguchi-kamicho, Itabashi-ku, Tokyo 173-8610, Japan

Table S1. Reproducibility of reporter gene assays

Compound	LXRα [1]	LXRβ [1]	AR [1]	GR [1]
**6**	290 ± 81 (13)[2]	130 ± 30 (12)[2]		
RU486				2.0 ± 0.48 (10)
OHF			170 ± 32 (11)	
**7**	19000 ± 390 (4)	15000 ± 200 (2)	17000	>20000
**8**	17000 ± 930 (4)	16000 ± 2300 (2)	11000	>20000
**9**	17000 ± 1200 (4)	16000 ± 2200 (2)	19000	19000 ± 50 (2)
**10**	>20000	>20000	17000	>20000
**11**	>20000	>20000	9600	>20000
**12**	8400 ± 370 (4)	3700 ± 1400 (3)	1800 ± 50 (2)	7400 ± 500 (2)
**1**			100	>20000
**13**			500	>20000
**14**	14000 ± 3600 (4)	6600 ± 430 (3)	1700	>20000
**15**	12000 ± 1300 (4)	6900 ± 1900 (3)	5600	17000 ± 750 (2)
**16**	16000 ± 2300 (4)	10000 ± 120 (2)	4000	>20000
**17**	>20000	>20000	15000	>20000
**18**	>20000	>20000	>20000	>20000
**19**	8400 ± 2400 (2)	9200 ± 650 (2)	1100	1300 ± 250 (2)
**20**	6900 ± 700 (2)	5500 ± 500 (2)	2400	3900 ± 650 (2)
**21**	11000 ± 2100 (2)	12000 ± 1100 (2)	9400	14000 ± 1100 (2)
**22**	12000 ± 3200 (2)	11000 ± 200 (2)	1000	8200 ± 100 (2)
**23**	7000 ± 1300 (2)	2400 ± 300 (2)	>20000	10000 ± 400 (2)
**24**	4600 ± 1300 (2)	4300 ± 450 (2)	>20000	>20000
**25**	8500 ± 610 (4)	9600	6100	>20000
**26**	>20000	>20000	540	760 ± 75 (2)
**27**	17000 ± 1600 (5)	18000 ± 650 (2)	580	1700 ± 0 (2)
**28**	>10000	>10000	1700	5200 ± 200 (2)
**29**	3200	3200	4600	7900
**30**	>20000	>20000	>20000	>20000
**31**	8800	8900	1600	17000
**32**	3100 ± 100 (2)	4600 ± 1200 (2)	390 ± 30 (2)	6900 ± 300 (2)
**33**	3100	3100	460	9800
**34**	7900	6000	680	>20000
**35**	6400	5400	920	7000
**36**	12000 ± 2900 (6)	3000 ± 530 (3)	6400	16000 ± 100 (2)
**2**	9200 ± 3000 (7)	880 ± 180 (7)	5200	10000 ± 1800 (2)
**37**	7100 ± 1200 (6)	4400 ± 800 (3)	>20000	>20000
**38**	7000 ± 600 (4)	5500 ± 160 (3)	>3000	>20000
**39**	>20000	>20000	>20000	>20000
**40**	>20000	>20000	>20000	>20000
**41**	>20000	>20000	14000	>20000
**42**	>20000	20000	5400	16000
**43**	5300 ± 850 (2)	>20000	59 ± 9.5 (2)	>20000
**44**[3]	>20000	>20000	1700 ± 100 (2)	>20000
**3**[3]	>20000	>20000	300	>20000

[1] Antagonistic activity: mean IC_{50} value (nM) ± SEM from 1 or *N* times independent experiments. [2] Agonistic activity: mean EC_{50} value (nM) ± SEM from *N* times independent experiments..
